# Pathogenicity of the Entomopathogenic Fungus *Metarhizium anisopliae* Var. *Major* on Different Stages of the Sunn Pest *Eurygaster integriceps*

**DOI:** 10.1673/031.013.15001

**Published:** 2013-12-13

**Authors:** Neda Sedighi, Habib Abbasipour, Hassan Askary, Aziz Sheikhi Gorjan, Jaber Karimi

**Affiliations:** 1 Department of Plant Protection, Faculty of Agricultural Sciences, Shahed University, Tehran, Iran; 2 Department of Biological Control, Iranian Research Institute of Plant Protection, Tehran, Iran; 3 Department of Pesticides, Iranian Research Institute of Plant Protection, Tehran, Iran

**Keywords:** LC_50_, LT_50_

## Abstract

The sunn pest, *Eurygaster integriceps* Puton (Hemiptera: Scutelleridae), is the most important insect pest of wheat and barley. The population management of this pest is of major concern to wheat producers. One of the potential control strategies is to use entomopathogenic fungi. This study evaluates the pathogenicity of the fungus *Metarhizium anisopliae* var. *major* (Metchnikoff) Sorokin (Hypocreales: Clavicipitaceae) on the sunn pest, *E. integriceps.* Five concentrations of the fungus were utilized, ranging from 1×10^4^ to 1×10^8^ conidia/mL, accompanied by controls. Fifth instar nymphs and adults (a migratory summer population and a diapausing population) previously exposed to fungi were sown to isolate the fungi, and the growth parameters were analyzed. A direct spray technique was used to expose the isolates to the *E. integriceps.* The experiment was repeated four times, and mortalities of the insects for all treatments were recorded daily. The results showed that the mortality of infected nymphs was significantly higher than the mortality of control nymphs. Also, the longevity of infected adults was lower than the controls. The results also showed that diapausing adults of the sunn pest were much more susceptible to infection than the summer adults. Estimated LC_50_ values for the M_14_ isolate were 1.4 × 10^6^, 1.4 ×10 ^5^ , and 2.3 × 10^3^ spores/mL against the aestivation population, the diapausing population, and 5^th^ instar nymphs, respectively. Estimated LT_50_ values using 10^8^ spores/mL of the Mm isolate on the aestivation and diapausing populations were 11.9 and 5.11 days, respectively. The results demonstrated that *M. anisoplaie* was effective on all of stages of *E. integriceps.* In addition, the nymphal stage was more susceptible than the adults.

## Introduction

In the regions of Central and West Asia and North Africa, the human population is steadily rising, while rainfall and arable land are limited, and a food security crisis is looming (El-Beltagy 2000). Cereals are an extremely important food commodity in most countries in this region, and wheat, *Tri ti cum aestivum* L., provides over 40% of the per capita dietary supply of calories and protein (FAO 2013). Wheat and barley, *Hordeum vulgare* L., are attacked by several species of pests. The sunn pest, *Eurygaster integriceps* Puton (Hemiptera: Scutelleridae), is the most damaging and economically important pest that predominantly attacks these plants, feeding on the leaves, stems, and grains, reducing yield, and injecting chemicals into the grain that destroy the gluten and greatly reduce the baking quality of the resulting flour (Javahery 1995; Hariri et al. 2000; Parker et al. 2003).

*Eurygaster integriceps* lives for one year and is univoltine (produces one generation annually). Around three months are spent actively on graminaceous plants. The rest of the year is spent overwintering in vegetation and litter and undergoing diapause, which includes two phases: aestivation during the hot, dry months of late summer and autumn and diapause during the cold winter months. In the southern area of its range (Iran, Iraq, and Turkey), *E. integriceps* overwinters at altitudes between 1000 and 2000 m a.s.l. As soon as temperatures rise above ˜12° C, they migrate back into lower plains, where the crops are (Critchley 1998). The pest affects around 15 million ha annually (Moore and Edgington 2006). There are many different methods for controlling *E. integriceps.* However, all the methods are to control its outbreak and not to eradicate it. The methods utilized for this purpose inelude chemical, biological, mechanical, and cultural control.

One of the research priorities identified by the Food and Agriculture Organization of the United Nations was to evaluate the potential of naturally occurring pathogens in *E. integriceps* overwintering sites (Jordan and Pascoe 1996). Entomoparasitic fungi (e.g., *Beauveria bassiana)* have demonstrated their potential to kill bugs when other biological agents do not, i.e., during diapause (Parker et al. 2003; El Bouhssini et al. 2004). Entomopathogenic fungi such as *B. bassiana* and *Metarrhizium anisopliae* (Metchnikoff) Sorokin (Hypocreales: Clavicipitaceae) have shown great potential for managing various insect pests (Inglis et al. 2001). In determining the effectiveness of introducing fungi to the overwintering localities of *E. integriceps,* scientists recorded significantly increased mortality in plots treated with *B. bassiana* and *M. anisopliae* compared to control plots (Parker et al. 2003). *Metarrhizium anisopliae,* the agents of the green muscardin disease, attach their spores into the epidermis of the insects. Then they pierce the epidermis, and after germinating they infiltrate the insect. This entomopathogenic fungus penetrates the haemolymph of the insect, grows and proliferates inside the body of the host, interacts with the insect's defense mechanisms, and finally sporulates on the host cadaver (Hajek and St. Leger 1994; Sedighi 2011).

The objectives of this study were to: (1) calculate the LC_50_ and LT_50_ of Iranian *M. anisopliae* isolates from different sources and regions, (2) select the most virulent isolate, and (3) compare the susceptibility of different developmental stages (nymphal and migratory summer and diapausing adults) of *E. integriceps* to infection by the entomopathogenic fungus M *anisopliae.*

## Materials and Methods

### Maintenance of field-collected insects

Adult *E. integriceps* were collected from their resting sites in the Varamin region (Tehran, Iran) in December 2009 (diapausing population) and in September 2009 (migratory summer population). The samples were maintained at 25 ± 2° C, 60% RH, and a 16:8 L:D photoperiod on wheat seeds. A piece of cotton soaked with water was used as a water source. The insects were kept for one week at these conditions, and dead insects were removed before the bioassays. The *E. integriceps* 5^th^ instar nymphs were collected from wheat fields of the Varamin region. Nymphs were kept under similar conditions as adults.

### Fungal isolates

The isolates of *M. anisopliae* var. *major* that were used in the experiments are now preserved at the Department of Agricultural Entomology, Iranian Research Institute of Plant Protection, Tehran, Iran (Table 1). Fungi were cultured on Sabouraud dextrose agar media and maintained at 23 ± 2° C and a 16:8 L:D photoperiod and were prepared using a two-step method. In this method, the hyphae were produced in a shaking Erlenmeyer flask and were then transferred to solid medium to produce conidia.

### Biphasic production: liquid phase

A liquid stage in the production system encourages rapid mycelial growth of the fungal culture, which can then be used to inoculate the second, solid stage in the production process. The liquid medium used in the first stage of production contained potato extract (200 g in 1000 mL distilled water), which is essential for growth. The liquid media were autoclaved in propylene bags at 121° C for 20 min (Jenkins et al. 1998). The 1 L Erlenmeyer flasks containing 250 mL of liquid media were incubated with spores of *M. anisopliae* in a shaking incubator (US-848DSRNL, Vision Scientific, www.visionbionex.com) at 100 rpm and 24° C for five days.

### Biphasic production: solid phase

The grain (300 g barley) was added to boiling distilled water (700 mL) and allowed to parboil for 1 hr. After cooling, it was autoclaved in propylene bags at 1 atm and 120° C for 1 hr and then transferred to a laminar flow cabinet. The liquid culture was diluted by 50% with sterile cold water, and the resulting liquid inoculum was added to the bags (150 mL/kg barley) (Jenkins et al. 1998). Conidia were harvested from 14-day-old sporulating cultures by scraping the surface of the barley with a spatula and suspending the conidia in sterile 0.04% aqueous Tween 80 (Merck, www.merck.com). Different concentrations of spores were prepared as required after several preliminary tests.

### Bioassay: adults

Batches of 10 *E. integriceps* adults were placed on filter paper in dishes (5 × 10 × 16 cm). Spore suspensions (4 mL) of *M. an-isopliae* were sprayed by a hydrolic handle sprayer, con-di nozzle on adults at different concentrations (10^4^, 10^5^, 10^6^, 10^7^, 10^8^, and 10^9^ conidia/mL). Dishes were incubated at 25° C, 75 ± 5% RH, and under a 16:8 L:D photoperiod. Control adults were sprayed with 4 mL of sterile distilled water containing 0.04% Tween 80. Dead insects were counted daily for 22 and 13 days for the migratory summer population and the diapausing population, respectively. Dead insects were surface sterilized by immersion in a solution of 50% sodium hypochlorite for 2 min, rinsed once in 70% ethanol and three times in sterile distilled water, and then placed on sterile Petri dishes with wet cotton and incubated at 25° C, 75 ± 5% RH to grow fungus on cadavers and to confirm infection with fungus. In total, 280 engorged adults were treated with various concentrations of the different strains.

### Bioassay: nymphs

The effects of the isolates M_i4_, IRAN 437_c_, and IRAN 715_c_ on the 5^th^ instar nymphs were studied. Spore suspensions (3 mL) of *M. an-isopliae* were sprayed on nymphs at different concentrations (10^3^, 10^4^,10^5^, 10^6^, 10^7^, and 10^8^ conidia/mL). Nymphs were maintained at 25 ± 2° C, 75 ± 5% RH, and under a 16:8 L:D photoperiod on wheat grains in plexiglass dishes (5×10×16 cm). There were 10 nymphs in each treatment. For each treatment dose, four replicates were used. Control nymphs were sprayed with 3 mL of sterile distilled water containing 0.04% Tween 80. Nymphs were monitored for seven days, and mortality was determined by larval wasting and immobility.

### Statistical analysis

Mortality was corrected using Abbott's formula (Abbott 1925). All experiments were assigned using completely randomized basic factorial design. Values of LC_50_, LC_99_, and 95% fiducial limits were calculated using Probit analysis (SAS Institute 2004). Graphs were plotted using Microsoft Excel software (www.microsoft.com).

## Results

**Figure 1.**
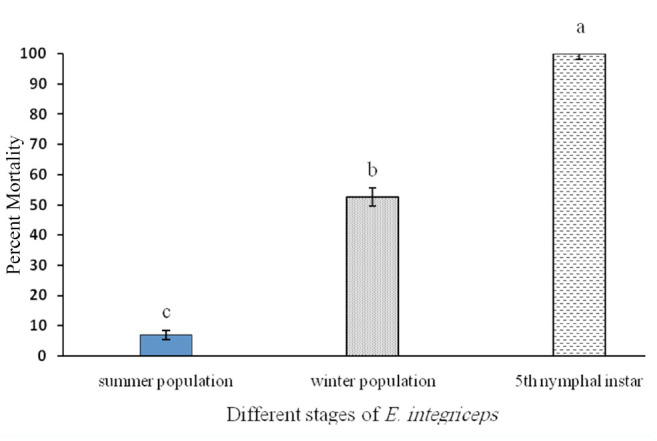
Comparison of insect mortality of three different stages of *Eurygaster integriceps* after six days when inoculated with conidial suspensions *of Meta rh i zi um anisopliae* var. *major.* High quality figures are available online.

All three studied isolates of *M. anisopliae* were pathogenic to *E. integriceps* (Table 2). Variations in their pathogenicity were noticed. Estimated LC50 values for the Iran 715_c_ and Iran 437_c_ isolates on 5^th^ instar nymphs were 1.4 × 10^3^ and 2.3 × 10^3^ conidia/mL, respectively. In addition, for the summer adults the M_14_, IRAN 437_c_, and IRAN 715_c_ values were 1.4 × 10^6^, 3.2 × 10^6^, and 4.3 × 10^7^ conidia/mL and for the winter adults they were 6.5 × 10^4^, 7.1 × 10^4^, and 3.6 × 10^5^ conidia/mL, respectively. The mortality of *E. integriceps* upon M_14_ treatment was the highest (58% and 92.5% after 10 days for summer and winter adults, respectively) when compared to treatments with the other two fungal strains (Table 3). The susceptibility of the nympal stage of *E. integriceps* to local strains of *M. anisopliae* is shown in Table 4. The times at which 50% lethality occurred (LT_50_ for 10^8^ conidia/mL) for the M_14_, Iran715_c_, and Iran 437_c_ isolates were 5.11, 6.31, and 9 days (diapausing adults) and 11.9, 17.52, and 29.64 days (summer adults), respectively. For the 5^th^ instar nymphs, these values were 3.45 and 3.12 days for the Iran715_c_ and Iran 437_c_ isolates, respectively (Table 5). Strain Iran 437_c_ was less virulent to the adult stages of *E. integriceps* in comparison with other strains. Nymphs were highly susceptible to all the isolates (Table 4). Isolates Iran 437_c_ and Iran 715_c_ caused 32% and 95% and 43% and 100% mortality in 5^th^ instar nymphs on days three and five, respectively, and these values reached 100% by day eight. The nymphs that were inoculated but not killed were not able to emerge as adults. The longevity of the adults was significantly affected, being shorter after pest treatment. The most susceptible stage was the nymphal stage, followed by the diapausing and summer population (Figure 1).

**Figure 2.**
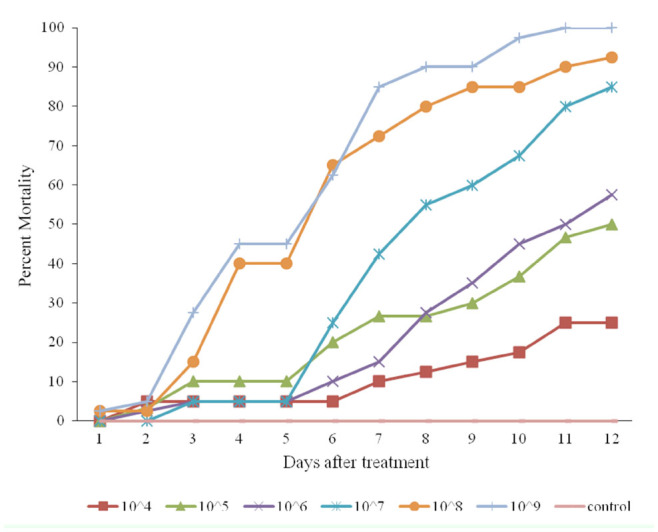
Cumulative mortality of *Eurygaster integriceps* (migratory and diapausing populations) after using a fungal bioassay with different concentrations of isolate M14 for I 2 days. High quality figures are available online.

**Figure 3.**
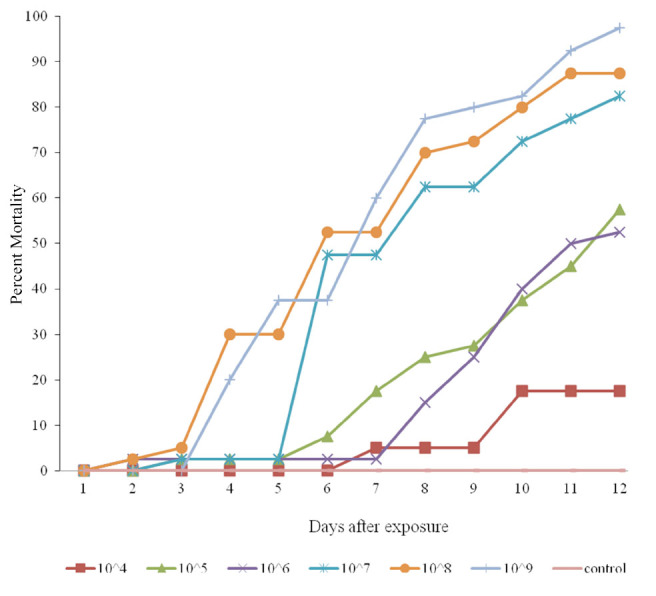
Cumulative mortality of *Eurygaster integriceps* (migratory and diapausing populations) after using a fungal bioassay with different concentrations of isolate IRAN 715_c_ for 12 days. High quality figures are available online.

**Figure 4.**
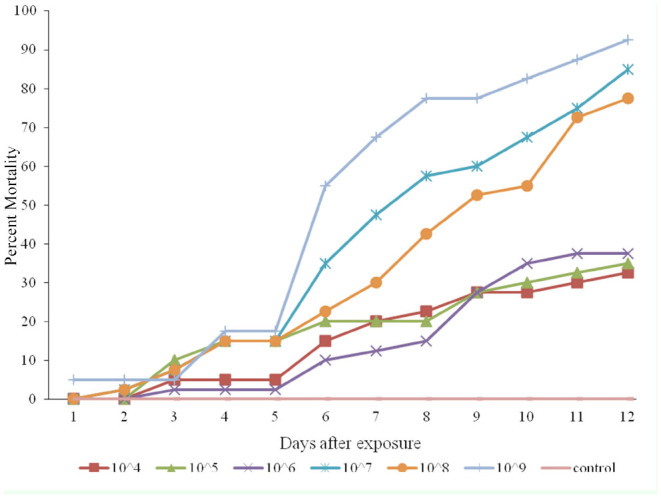
Cumulative mortality of *Eurygaster integriceps* (migratory and diapausing populations) after using a fungal bioassay with different concentrations of isolate IRAN 437_c_for 12 days. High quality figures are available online.

The percentage of cumulative mortality in the diapausing population treated with the Mi_4_ strain of *M. anisopliae* increased proportion-ately with increasing concentrations of the test substance, as 25%, 57%, 85%, 92%, and 100% cumulative mortality was reported in the 10^4^, 10^6^, 10^7^, 10^8^, and 10^9^ spore/mL treatments, respectively (Figure 2). Similar results were found for the other strains (Figures 3 and 4). Insects that died within the first 24 hours were not considered in the analysis, because fungus needs more than 24 hours to affect an insect.

When the treated insects died, they showed typical symptoms of infection and were covered with fungus after incubating in an initially sterile Petri dish in conditions with high humidity. Infected bodies of nymphs and adults of *E. integriceps* also turned hard and dry. The fungal outgrowth and sporulation on treated nymphs and adults were observed for multiple days after treatment. At first the color of the fungal outgrowth and sporulation, which was observed on the surface of pests' cadavers, was white. After some days, the color of the fungus slowly changed to green.

Mortality rates of different growth stages of *E. integriceps* were significantly different. Average mortality after treatment with fungi suspension on the 5^th^ instar nymphs was 100% after six days of spraying, while the average mortality percentages for the diapausing and summer adults were calculated at 54.58% and 52.50%, respectively, after 12 days.

## Discussion

*Eurygaster integriceps* has been reported to be susceptible to infection by several fungi in overwintering sites (Parker et al. 2003). Some of these naturally infecting pathogens, like *B. bassiana* and *M. anisopliae*, are among the most virulent pathogens to the pest. *Metarhizinm anisopliae* causes the highest mortality rate for *E. integriceps.* Parker et al. (2003) suggested the use of this entomopathogenic fungus as a biological control agent for management of *E. integriceps* on wheat fields and in overwintering sites. The results of our study revealed that isolate Mi_4_ was more effective than the Iran 437_c_ and Iran 715_c_ isolates at combating *E. integriceps.*

Two previous studies on the efficacy of M *anisopliae* on *E. integriceps* were considered. One study (Bandani et al. 2006) indicated that the LC_50_ values of two isolates (Ml 89 and 4556) of *M. anisopliae* on adults (with an immersion method) were 7.704 × 10^4^ and 3.38 x 10^5^ conidia/mL, respectively. The LT_50_ values for 1 × 10^8^ and 1 × 10^9^ spores/mL test suspensions for Ml89 and 4556 isolates were 14.8 and 11.2 days, respectively. The other study (Kivan 2006) was a laboratory pathogenicity experiment on the effects of isolate 3540 M *anisopliae* from *Gallería mellonella* on adult *E. integriceps.* In a single exposure concentration (1 × 10^6^ conidia/mL) assay, the adults were immersed in 10 mL of a fungal suspension. The mortality was 100% in *M. anisopliae* at eight days after treatment. In our study, the estimated LC_50_ values for each isolate (M_14_, Iran715_c_, and Iran 437_c_) for the summer adults were 1.4 × 10^6^, 3.2 × 10^6^, and 4.3 × 10^7^ conidia/mL, and for the diapausing adults they were 6.5 × 10^4^, 7.1 × 10^4^, and 3.6 ×10^6^ conidia/mL, respectively. The results of our study showed that different isolates of *M. anisopliae* had different effects on *E. integriceps* adults. So, identification, application, and screening of different isolates in bioassays will provide promising potential for finding efficient isolates to be used in field studies as bio-pesticides. Generally, direct immersion of the insects in fungal suspensions resulted in high percentages of overall mortality (Kivan 2006). Our study showed that the probability of placing conidia on the insect is higher when using the direct immersion method than when spraying, but the direct immersion method is not applicable in the field.

The results from the insect bioassays clearly indicate that *E. integriceps* is susceptible to infection by *M. anisoplaie.* However, comparing the LC_50_ values showed differences in the mortality rates at different stages of life, as the nymphal stages were more susceptible than the adult populations. Different developmental stages of insects have different sensitivities to fungi, so to most effectively apply fungi it is useful to know the developmental stage of the insect being treated.

In this study, the effects of fungal isolates were evaluated on the various nymphal and adult stages of *E. integriceps* so that they can be used in the field against the most susceptible stages. Because hardness and resistance of the insect cuticle has an important role in incidence of fungi infections, susceptibility of nymphal stages could be attributed to their reduced cuticle thickness (Ghazavi 2002). There were also significant differences between diapausing and summer adult populations. Diapausing adults of *E. integriceps* showed more susceptibility than summer populations. Reduction of food leads to increased pest sensitivity to fungi, and conversely, adequate feeding could reduce infection efficiency (Sarami and Zand 2003). So, insects in the diapausing stage, due to lack of nutrition and a reduction in the amount of reserved energy (fat) in the their bodies, will be more susceptible than insects in the summer population. In their experiments on winter adults of *E. integriceps* in hibernating regions and wheat fields, Moore and Edgington (2006) concluded that because of exposure to cold, wet soil, and a lack of nutrition during the four months, winter *E. integriceps* adults were weaker and were faced with energy shortages, thus providing better conditions for infection by pathogenic fungi. So, application of fungi in the wintering sites of *E. integriceps* before they move to the wheat fields was recommended. These results are consistent with the results of our study, and the following characteristics can be attributed to the diapausing stage: 1) reduced food storage, 2) weakness due to more varied environmental conditions, and 3) reduced body fat.

**Table 1.**
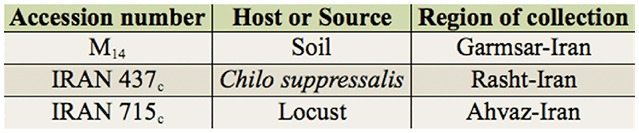
Isolates of *Metarhizium anisopliae* var. *major* used in the experiments with *Eurygaster integriceps.*

**Table 2.**
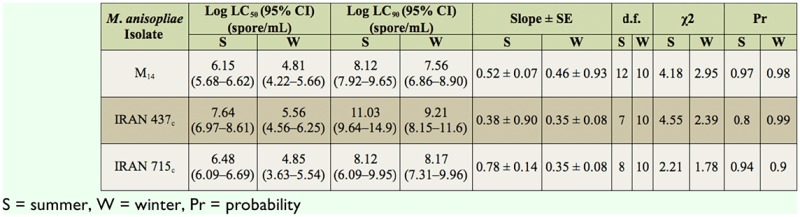
Estimated LC_50_ values of isolates of IRAN 71 5_c_, Iran 437_c_, and Mk of *Metarhizium anisopliae* var. *major* on summer and winter populations of *Eurygaster integriceps* at *22* and I *2* days post-exposure, respectively.

**Table 3.**
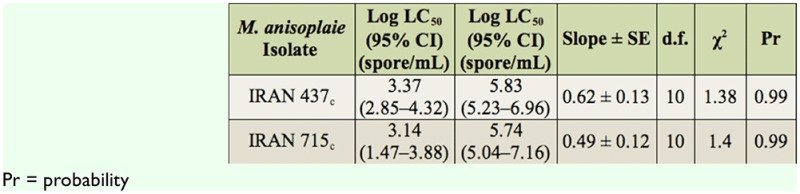
Estimated LC_50_ values of isolates of IRAN 715_c_ and Iran 437_c_ of *Metarhizium*. *anisoplaie* var. *major* for the 5^th^ instar nymphs of *Eurygaster integriceps* after six days.

**Table 4.**
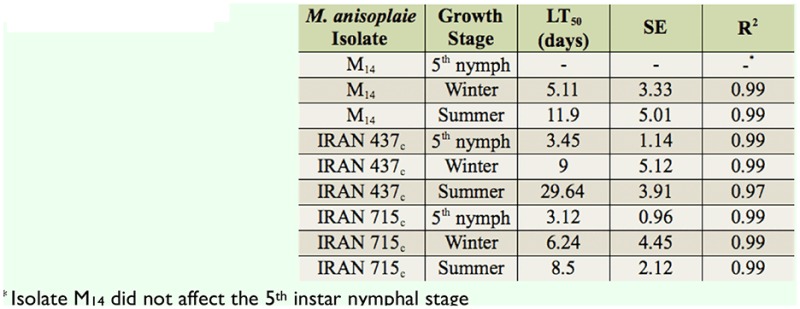
Estimated LT_50_ values for different isolates of *Metarhizium anisoplaie* var. *major* at I 0^8^ conidia/mL on different stages of *Eurygaster integriceps.*
